# Promoted Hole Transport Capability by Improving Lateral Current Spreading for High‐Efficiency Quantum Dot Light‐Emitting Diodes

**DOI:** 10.1002/advs.202001760

**Published:** 2020-11-01

**Authors:** Qianqian Wu, Fan Cao, Haoran Wang, Jianquan Kou, Zi‐Hui Zhang, Xuyong Yang

**Affiliations:** ^1^ Key Laboratory of Advanced Display and System Applications of Ministry of Education Shanghai University 149 Yanchang Road Shanghai 200072 China; ^2^ State Key Laboratory of Reliability and Intelligence of Electrical Equipment Hebei University of Technology 5340 Xiping Road, Beichen District Tianjin 300401 China

**Keywords:** current spreading, electroluminescence, hole transport capability, light‐emitting diodes, quantum dots

## Abstract

Carrier imbalance resulting from stronger electron injection from ZnO into quantum‐dot (QD) emissive layer than hole injection is one critical issue that constrains the performance of QDs‐based light‐emitting diodes (QLEDs). This study reports highly efficient inverted QLEDs enabled by periodic insertion of MoO_3_ into (4,4′‐bis(*N*‐carbazolyl)‐1,1′‐biphenyl) (CBP) hole transport layer (HTL). The periodic ultrathin MoO_3_/CBP‐stacked HTL results in improved lateral current spreading for the QLEDs, which significantly relieves the crowding of holes and thus enhances hole transport capability across the CBP in QLEDs. Comprehensive analysis on the photoelectric properties of devices shows that the optimal thickness for MoO_3_ interlayer inserted in CBP is only ≈1 nm. The resulting devices with periodic two insertion layers of MoO_3_ into CBP exhibit better performance compared with the CBP‐only ones, such that the peak current efficiency is 88.7 cd A^−1^ corresponding to the external quantum efficiency of 20.6%. Furthermore, the resulting QLEDs show an operational lifetime almost 2.5 times longer compared to CBP‐only devices.

## Graphical abstract

This study reports over 20% external quantum efficiency quantum dot light‐emitting diodes with inverted structure that are based on periodic insertion of MoO_3_ into a (4,4’‐bis(*N*‐carbazolyl)‐1,1’‐biphenyl) (CBP) hole transport layer (HTL). The periodic MoO_3_/CBP‐stacked HTL results in improved current spreading, which significantly enhances hole transport capability across the CBP and thus facilitates charge injection balance in devices.

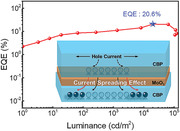

## 1 Introduction

Featured with excellent properties of narrow emission linewidth, emission wavelength tunability, and superior solution processability, quantum dots (QDs)‐based light‐emitting diodes (QLEDs) have become one of the most potential candidates for next‐generation display and solid‐state lighting technologies.^[^ ^1^, ^2^, ^3^, ^4^ ^]^ In the past 25 years, intensive investigations on QLEDs have significantly upgraded their external quantum efficiencies (EQEs) ranging from less than 0.01 up to over 20%, which makes QLEDs competitive with fluorescent organic LEDs.^[^ ^5^, ^6^, ^7^, ^8^, ^9^, ^10^, ^11^, ^12^, ^13^ ^]^ However, the imbalance for carriers injected into the QD layer in QLEDs remains a concern, which prevents the performance from being further promoted. The carrier imbalance is attributed to the energy level mismatch between QDs layer and carrier transport layer (CTL). In addition, traditional electron transport layer (ETL) such as ZnO has higher carrier mobility than most hole transport materials, and thus leads to imbalanced carrier injection capability.^[^ ^14^ ^]^ The higher electron mobility results in the accumulation of excess electrons on QDs layer and hole transport layer (HTL), thus facilitating nonradiative Auger recombination by QD charging and ultimately gives rise to the low efficiency of devices.^[^ ^15^, ^16^, ^17^ ^]^ To resolve these problems, researchers focus on reducing electron injection to achieve balanced charge in devices. Inserting charge buffer layers such as poly(methyl methacrylate) (PMMA), polyethylenimine (PEI), Al_2_O_3_, and Cs_2_CO_3_ as electron blocking layers between the QDs and ETL is the most common method to balance charge injection and thus gain better device efficiency.^[^ ^18^, ^19^, ^20^, ^21^ ^]^ However, improvements in the performance for these structural devices have been achieved with the cost of suppressing electrons and increasing system resistance.

A better alternative approach for improving device performance is enhancing the hole injection/transport ability to promote the charge balance. Doping metal oxides materials such as MoO_3_, V_2_O_5_, or WO_3_ in organic HTLs has been proven that the co‐doping approach can efficiently regulate the hole transport of semiconductors.^[^ ^22^, ^23^, ^24^, ^25^ ^]^ However, co‐evaporation is not only troublesome to operate, but also doping heavy metal ions in the HTL adjacent to the QDs will quench the fluorescence of QDs.^[^ ^26^, ^27^, ^28^ ^]^

In this work, we experimentally and numerically improve the hole transporting capability by periodically inserting ultrathin MoO_3_ in (4,4′‐bis(*N*‐carbazolyl)‐1,1′‐biphenyl) (CBP) and investigate the mechanism for the improved hole transporting capability in QLEDs. Thin MoO_3_ layer in CBP allows holes to tunnel through the spacer, thereby leading to improved lateral current spreading, which not only avoids current crowding, but also improves the hole transport capability of CBP film, thereby improving charge injection balance in QLEDs and realizing better device performance.

## 2 Results and Discussion

**Figure 1:**
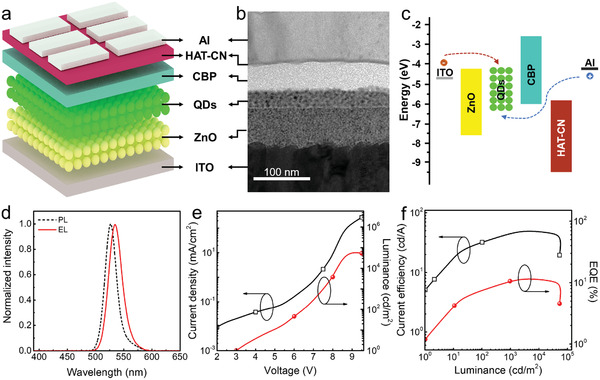
a) Schematic device structure, b) cross‐sectional TEM image, and c) energy band diagram in the unbiased condition of the control device. d) Normalized EL/PL spectra, e) *J–V–L*, and f) *CE–L–EQE* characteristics for the control device.

A schematic diagram of our inverted QLED consists of indium tin oxide (ITO) cathode, ZnO ETL, QDs emissive layer (EML), CBP HTL, 1,4,5,8,9,11‐hexaazatriphenylene hexacarbonitrile (HAT‐CN) hole injection layer (HIL), and Al anode as a control device (**Figure** **1**a). All functional layers are complete and compact after multilayer film stacking, a cross‐sectional transmission electron microscopy (TEM) of the above control device is demonstrated in Figure 1b. ZnO nanoparticles (NPs) serve as ETL owing to their high carrier mobility (≈1.8 × 10^−3^ cm^2^ V^−1^ s^−1^) and matched conduction band minimum with that of QDs.^[^ ^18^ ^]^ HAT‐CN is chosen as the HIL because the HAT‐CN HIL in the inverted device can form ohmic hole injection.^[^ ^29^ ^]^ The energy band diagram of the control device is shown in Figure 1c.^[^ ^30^, ^31^ ^]^ Due to the relatively small energy barrier between ZnO NPs and QDs, electrons can be easily injected into QD layer. As shown in Figure 1d, compared to the normalized photoluminescence (PL) of QDs, the normalized electroluminescence (EL) emission peaking at 534 nm has a red shift of ≈8 nm. The wavelength shift arises from the Stark effect induced by electric field and interaction of QDs in film state.^[^ ^7^, ^32^ ^]^ The current density–voltage–luminance (*J–V–L*) characteristic shows that the maximum luminance of the device can reach 62 590 cd m^−2^ with a low turn‐on voltage (*V*_turn‐on_) of 3.0 V. The current efficiency–luminance–external quantum efficiency (*CE–L–EQE*) characteristic demonstrates that the device can achieve a high CE of 50.7 cd A^−1^ with the EQE of 11.8% at high luminance of 3775 cd m^−2^, indicating efficient carrier injection into QDs (Figure 1e,f).

**Figure 2:**
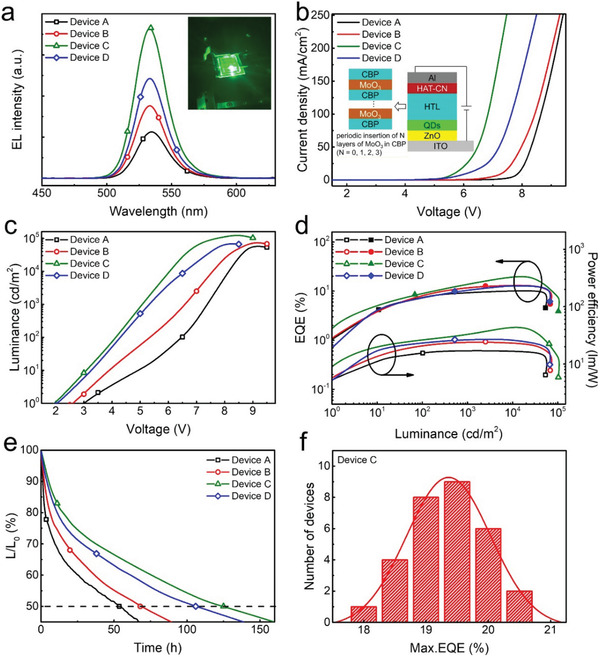
a) EL spectra for devices A–D at the current density of ≈90 mA cm^−2^. b) *J–V*, c) *L–V*, d) *EQE*–*L*–*PE*, and e) operating lifetime curves of devices A–D. f) Performance reproducibility of the device C. The photograph in (a) shows a working device C (with an emission area of 4 mm^2^ operated under 90 mA cm^−2^). The inset of (b) demonstrates the device structure based on periodically inserting *N* layers of MoO_3_ in CBP, for which *N* varies from 0 to 3 nm.

To further enhance the hole transport capability of CBP HTL, we devise a stacked multi‐layer HTL architecture by periodically inserting MoO_3_ thin film into CBP, and the best‐performing QLEDs with the HTL structure of CBP (50 nm) (device A), CBP (25 nm)/MoO_3_ (1 nm)/CBP (25 nm) (device B), CBP (16.7 nm)/MoO_3_ (1 nm)/CBP (16.7 nm)/MoO_3_ (1 nm)/CBP (16.7 nm) (device C), and CBP (12.5 nm)/MoO_3_ (1 nm)/CBP (12.5 nm)/MoO_3_ (1 nm)/CBP (12.5 nm)/MoO_3_ (1 nm)/CBP (12.5 nm) (device D) were fabricated and compared. **Figure** **2**a shows the EL spectra for devices A–D with a peak wavelength of 534 nm under the same current density of ≈90 mA cm^−2^. With the number of MoO_3_ insertion layer increases, the EL intensity exhibits a tendency to first increase and then gradually decrease with the variation of hole injection (Figure 2b). The inset in Figure 2a displays a corresponding photograph of device C. Figure 2b–e shows a comprehensive comparison on the *J–V*, *L–V*, *CE–L*, and power efficiency–luminance (*PE–L*) characteristics for these devices. With the number of periodically inserted MoO_3_ into CBP raising from 0 to 2, the current density rises gradually. However, further increase in the number of MoO_3_ layer causes a dramatic decrease in current density. This is also consistent with the *L–V* trend (Figure 2c). Benefiting from the enhancement of hole injection, the carrier recombination in QDs is more efficient and the *V*_turn‐on_ is accordingly reduced from 3 V to a low *V*_turn‐on_ of 2.0 V. Meanwhile, the maximum luminance of device C reaches 129 940 cd m^−2^, of which the value is much higher than 62 590 cd m^−2^ for device A, 79 110 cd m^−2^ for device B, and 71 320 cd m^−2^ for device D, respectively. The peak EQE of 20.6% and PE of 46.4 lm W^−1^ for device C are higher than the efficiency values (maximum EQE: 11.9% and maximum PE: 19.9 lm W^−1^) of the device without MoO_3_ inserted. The detailed performances for devices A–D are summarized in **Table** **1**. Achieving a low *V*_turn‐on_ is beneficial for long‐term QLEDs. Figure 2e shows that device C has a significantly longer operational lifetime than other devices, i.e., the *T*_50_ (taken as the time when the brightness reduced to L_0_/2) of device C tested in the air is as long as 125 h under the luminance of 100 cd m^−2^.^[^ ^18^ ^]^ However, those values of *T*_50_ for devices A, B, and D can only reach 54, 68, and 106 h at the same initial luminance, respectively. Figure 2f shows the histogram of the peak EQE values with 30 devices C. Both the high average peak EQE of 19.5% and the low relative standard deviation of 3.12% demonstrate good performance reproducibility. The EQE histogram of devices A, B, and D, and the average EQE and error ranges of devices A–D are shown in Figures S1 and S2 (Supporting Information), respectively. In addition, as a comparison, the device with a co‐evaporated CBP and MoO_3_ structure (ITO/ZnO/QDs/CBP:MoO_3_/HAT‐CN/Al) was also fabricated (Figure S3, Supporting Information). The luminance and EQE for the device are 70 500 cd m^−2^ and 7.3%, respectively, and these numbers are much lower than those of the device with periodically stacked HTL structure, which is mainly attributed to that the co‐evaporation process causes the emission quenching of QDs contacted with metal oxide.

**Table 1:** Summary of the detailed output parameters for devices A–D

Device	*V*_turn‐on_ [V]	Maximum luminance [cd m^−2^]	Maximum EQE [%]	Maximum CE [cd A^−1^]	Maximum PE [lm W^−1^]	Lifetime [h]
Device A	3.0	62 590	11.9	50.7	19.9	54
Device B	2.5	79 110	14.0	59.8	25.0	68
Device C	2.0	129 940	20.6	88.7	46.4	125
Device D	2.2	71 320	13.3	57.1	26.9	106

**Figure 3:**
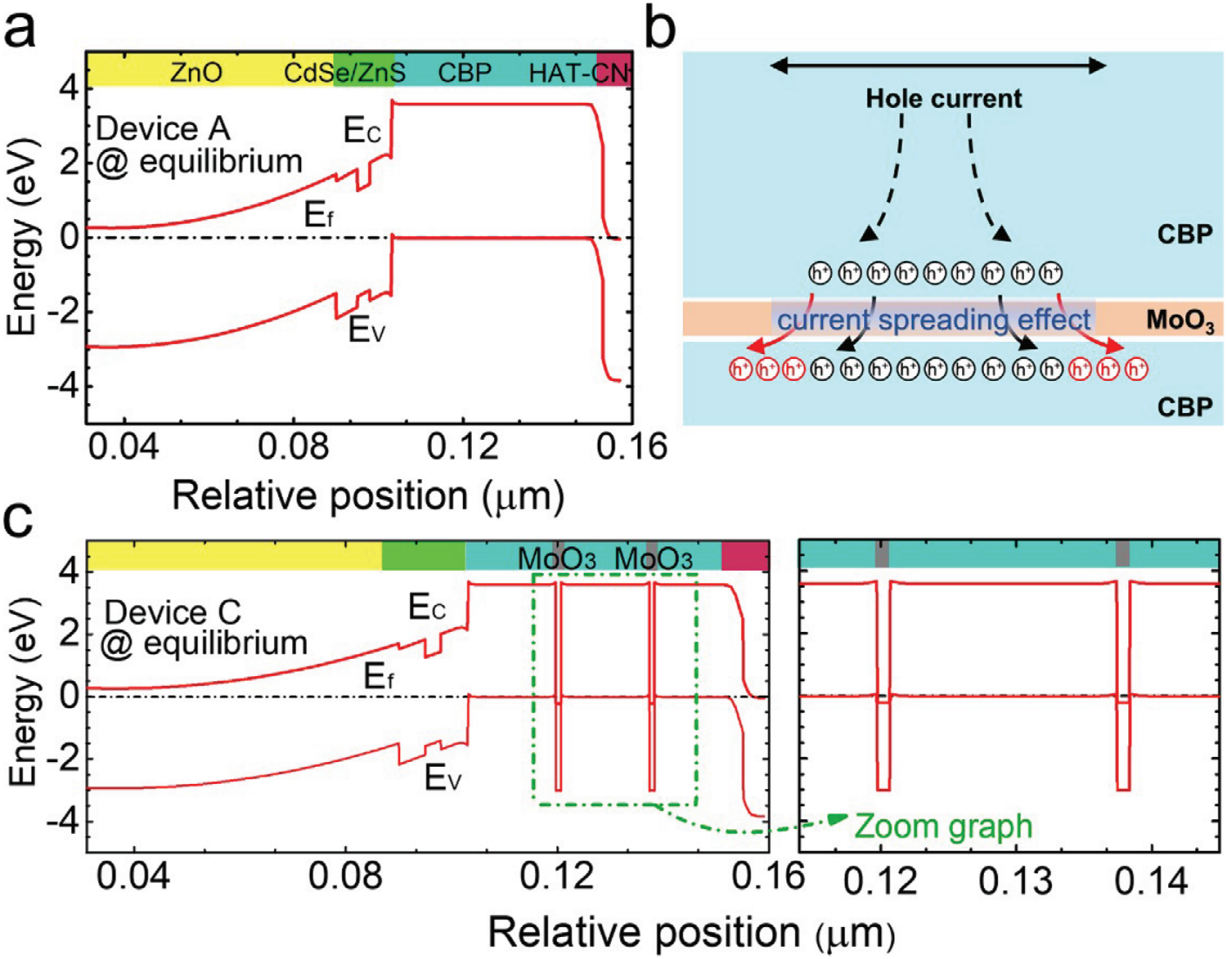
Energy band diagrams for a) device A at the equilibrium state. b) Schematic diagram of current spreading at the CBP/MoO_3_/CBP interface. c) Energy band diagrams for device C the equilibrium state. *E*_c_, *E*_v_, and *E*_f_ represent the conduction band, the valance band, and quasi‐Fermi level, respectively.

To further investigate the mechanisms on the improved hole transporting capability of HTL by periodically inserting MoO_3_ interlayers in CBP, we numerically calculate the energy level and current distribution for the QLEDs with different periodic numbers of MoO_3_ inserted into CBP. The energy band profiles for devices A–D at the equilibrium state are demonstrated in **Figure** **3** and Figure S4 (Supporting Information).

The current flows both laterally and vertically when the current is injected from the bonding metal (which can be also understood as contact between the ITO or Al and “wires”). The hole mobility is generally lower than that of electrons due to the relatively large effective mass for holes. Thus, the *p‐*type conductivity for the HTL will be poor. Moreover, the ratio of length/thickness of QLED device is very large (>5000:1), and the holes will experience no current spreading layer before being injection into the QD emissive layer (Figure 3a). As a result, very significant current crowding for holes will occur.^[^ ^33^, ^34^ ^]^ This further causes the asymmetry for the electron and hole concentrations in the QDs, thus limiting the luminous efficiency of QLEDs. According to Figure 3b,c, when inserting two layers of 1 nm thick MoO_3_ in the CBP HTL, the interband tunneling process occurs.^[^ ^35^ ^]^ The tunnel junction properly increases the vertical resistance for the HTL and helps to balance the electrical conductivity for the ETL and the HTL, which can improve the lateral current spreading in the HTL.^[^ ^36^ ^]^ Note that the tunnel junction has to be well optimized, otherwise a very large vertical resistance will appear, then altering the current spreading layer to the current blocking layer, which therefore can offset the advantage of the current spreading effect and hindering the carrier injection efficiency.

**Figure 4:**
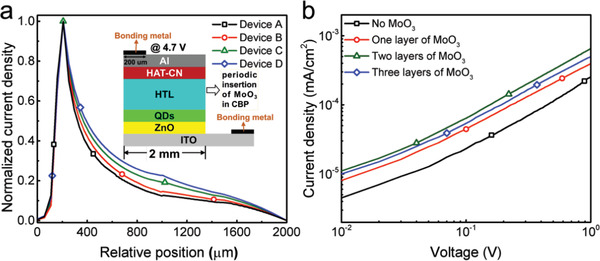
a) Calculated lateral current distribution in the CBP layer that is closest to QD emissive region for devices A–D at the voltage of 4.7 V, respectively. b) *J–V* curves for hole‐only devices.

To further probe the effects of the tunnel junction on improving the current spreading in QLEDs, we calculate and show the lateral current distribution in the CBP layer that is closest to the QD emitting region for the four devices in **Figure** **4**a. It is notable that the current density becomes more uniform as the number of the inserted MoO_3_ layer increases, and therefore device D has the most uniform current spreading due to the larger vertical resistivity of tunnel junction helping to modulate the current flow path.^[^ ^37^ ^]^

On the other hand, the film quality such as morphology and roughness are also crucial issue affecting QLED performance in terms of charge transport, especially for leakage current.^[^ ^38^ ^]^ Atomic force microscopy was carried out characterizing the film quality of CBP films without MoO_3_ and with two periodic MoO_3_ interlayers deposited on glass. There is no distinct difference in root mean square roughness of CBP films with and without the insertion of MoO_3_ interlayers (Figure S5, Supporting Information). Therefore, we can conclude that the difference in *J–V* curves (Figure 4a) is mainly due to the current spreading effect. Moreover, the enhanced current spreading of devices B, C and D facilitates the hole injection to QDs emitting region (Figure 4b), which has the same pattern with *J–V* curves from Figure 2b. Noticeably, the lower hole current density for device D than that of device C is attributed to that the more MoO_3_ interlayers in device D result in the accumulated stress at the interface and then thus increase the number of defects.^[^ ^39^, ^40^ ^]^

**Figure 5:**
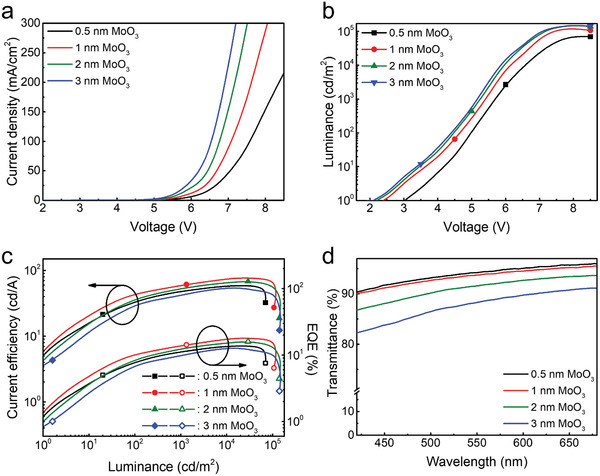
a) *J–V*, b) *L–V*, c) *CE–L–EQE*, and d) transmittance characteristics of the devices with an HTL structure of CBP (16.7 nm)/MoO_3_ (*x* nm)/CBP (16.7 nm)/MoO_3_ (*x* nm)/CBP (16.7 nm), for which *x* varies from 0.5 to 3 nm.

Different thicknesses of MoO_3_ inserted into CBP (CBP (16.7 nm)/MoO_3_ (*x* nm)/CBP (16.7 nm)/MoO_3_ (*x* nm)/CBP (16.7 nm), *x* = 0.5–3 nm) are also optimized for maximizing the hole injection into QD EML. From the *J–V* curve in **Figure** **5**a, we can see that when MoO_3_ becomes thicker, the current density of device is also increased simultaneously, which is accompanied by a higher device luminance (Figure 5b). This suggests that a thicker MoO_3_ interlayer in CBP HTL would greatly enhance hole injection and obtain better carrier balance. It is also noted that the maximum EQE of 20.6% is obtained when the MoO_3_ inserted CBP is 1 nm (Figure 5c) because of the reduced transmittance of HTLs as the thickness of MoO_3_ further increases (Figure 5d) and high current density‐induced device performance degradation such as electric field‐assisted dissociation of excitons, Coulombic degradation, and excessive heating at higher current density.^[^ ^41^, ^42^ ^]^

## 3 Conclusion

Inverted QLEDs with periodic MoO_3_ interlayer into CBP to improve current spreading for better hole injection capability and high device performance are demonstrated. The device utilizing periodical two insertion layers of 1 nm MoO_3_ within the CBP achieves the best performance (peak luminance of 129 940 cd m^−2^, EQE of 20.6%), and an almost 2.5‐fold device lifetime enhancement as compared to MoO_3_‐free QLED. The excellent performance is attributed to the periodic insertion of MoO_3_ into CBP, which induces tunnel junctions in the HTL and properly increases the vertical resistance, resulting in the increase in the lateral current spreading in QLEDs, which in turn significantly promotes the hole transport capability of HTL and balances charge in devices. These results indicate that the periodic HTL design for the CTL is an effective way to adjust electrical property, ultimately achieving high‐efficiency and stable QLEDs.

## 4 Experimental Section

##### Synthesis of ZnO NPs

ZnO NPs synthesis was based on previous methods to make some amendments.^[^ ^29^ ^]^ Briefly, tetramethylammonium hydroxide (5.5 mmol) dissolved in ethanol (10 mL) solution and zinc acetate (3 mmol) dissolved in dimethyl sulfoxide (30 mL) solution were blended and stirred in ambient temperature for 1 h. Then, the precipitation was centrifuged twice and dispersed in ethanol (30 mg mL^−1^).

##### Device Fabrication

QLEDs were fabricated by hybrid thermal evaporation/spin‐casting method, where the Al, CBP, and HAT‐CN layers were formed by thermal evaporation, and the others were formed through spin‐casting. The patterned ITO substrates were cleaned in deionized water, acetone, and ethanol for 30 min in ultrasonic bath, then cleaned for 20 min with O_2_‐plasma prior to transferring the substrate to a glove box for spin‐casting process. ZnO NPs (60 nm) were spun‐cast at 2000 rpm and subsequently annealed at 150 °C for 30 min. 18 mg mL^−1^ of green QDs (CdSe/ZnS QDs dissolved in octane, purchased from Mesolight Inc., 25 nm) was spun‐cast at 3000 rpm for 40 s with the annealing temperature of 90 °C for 20 min. CBP (50 nm), MoO_3_ (1 nm), HAT‐CN (4 nm) were sequentially precipitated in high‐vacuum evaporation chamber. Next, the HIL HAT‐CN was fully covered by the Al‐metal (100 nm) to form the reflective electrode and the area of the emitted light from the bottom of QLEDs was set to 2 mm × 2 mm.

##### Device Characterization

The cross‐sectional TEM of tandem QLEDs was measured by the dual‐beam focused ion beam (scanning electron microscope, Omniprobe AutoProbe 200.2 robot hand). The thicknesses of all solution‐processed layers were conducted by tencor alpha‐step500 step analyzer. Monitor of evaporation velocity and thicknesses of CBP, MoO_3_, HAT‐CN, and Al was via quartz crystal microbalance. The detailed characteristics of QLEDs were investigated by PR‐670 Spectra Colorimeter coupled with Keithley 2400 source meter. The half‐lifetime (*T*_50_) of devices was investigated through OLED aging lifespan test instrument (ZJZCL‐1).

##### Device Simulations

With the aid of Crosslight APSYS software,^[^ ^43^, ^44^ ^]^ the numerical calculations were also made to further reveal the underlying device physics of the improved hole injection capability for the investigated QLEDs (the key parameters involved in the simulations are listed in Table S1, Supporting Information). In these simulations, the interband optical transition model and the carrier transport model were established to calculate the energy level and current distribution based on the numerical solutions of quantum states, Schrödinger equations, Poisson's equation, the current continuity equation, and drift‐diffusion processes for carriers, which were consistent with appropriate boundary conditions. In addition, for obtaining the good agreement between the experimentally measured results and the numerically calculated ones, the nonradiative recombination of carriers was taken into consideration by using the Auger recombination and the Shockley–Read–Hall recombination models.^[^ ^45^, ^46^ ^]^ Moreover, the energy band gap and electron affinity for ZnO, CdSe/ZnS, CBP, MoO_3_, and HAT‐CN were also important parameters for the simulation.^[^ ^47^, ^48^ ^]^

## Conflict of Interest

The authors declare no conflict of interest.

## Supporting information

Supporting Information
