# Supplementary material for: Promoted Hole Transport Capability by Improving Lateral Current Spreading for High‐Efficiency Quantum Dot Light‐Emitting Diodes
Source: Adv Sci (Weinh). 2020 Nov 1;7(23):2001760. doi: 10.1002/advs.202001760 (PMC7709982; doi:10.1002/advs.202001760)
Supplement: Supplementary file 1 — Supporting Information [file ADVS-7-2001760-s001.pdf]

WILEY-VCH

© 2020 Wiley-VCH GmbH

ADVANCED SCIENCE
Open Access

Supporting Information

for *Adv. Sci.*, DOI: 10.1002/advs.202001760

Promoted Hole Transport Capability by Improving Lateral Current Spreading for High-efficiency Quantum Dot Light-Emitting Diodes

Qianqian Wu, Fan Cao, Haoran Wang, Jianquan Kou, Zi-Hui Zhang, and Xuyong Yang**

Supporting Information

Promoted Hole Transport Capability by Improving Lateral Current Spreading for High-efficiency Quantum Dot Light-Emitting Diodes

Qianqian Wu, Fan Cao, Haoran Wang, Jianquan Kou, Zi-Hui Zhang, and Xuyong Yang**

Q. Wu, F. Cao, H. Wang, Prof. X. Yang
Key Laboratory of Advanced Display and System Applications of Ministry of Education, Shanghai University, 149 Yanchang Road, Shanghai 200072, China
E-mail: yangxy@shu.edu.cn

J. Kou, Prof. Z.-H. Zhang
State Key Laboratory of Reliability and Intelligence of Electrical Equipment, Hebei University of Technology, 5340 Xiping Road, Beichen District, Tianjin 300401, China
E-mail: zh.zhang@hebut.edu.cn

SI-1. Histogram of peak EQEs measured from 30 devices of Device A, B and D.

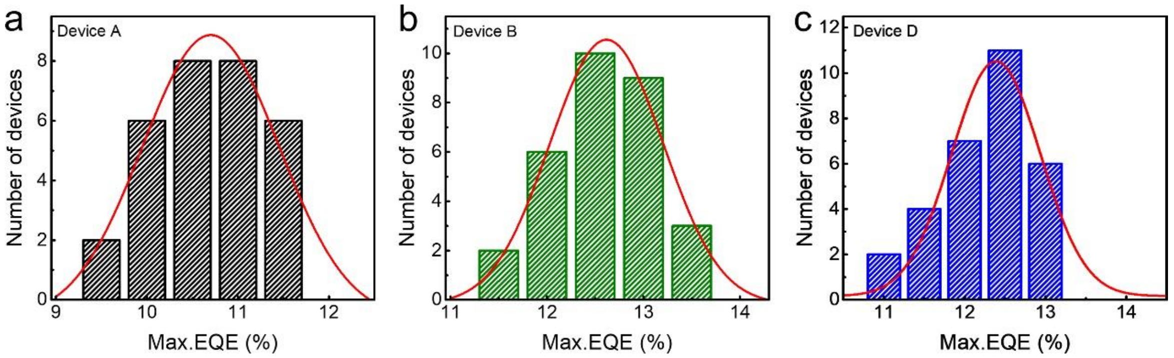

Figure S1. EQE reproducibility of (a) Device A, (b) Device B and (c) Device D. The average peak EQE of Device A, B and D are 10.9%, 12.8% and 12.4%, respectively, and the relative standard deviations of Device A, B and D are 5.08%, 4.28% and 3.49%, respectively, showing good performance reproducibility.

SI-2. Average EQE and error range curve of devices A-D.

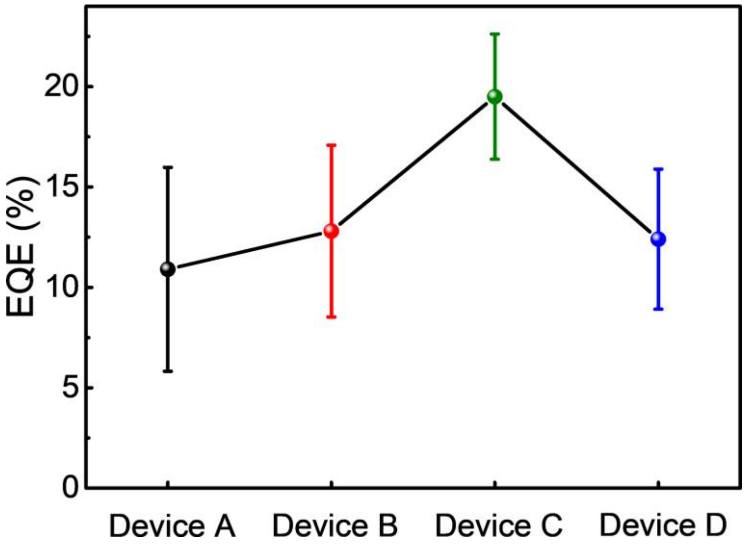

Figure S2. The average EQE and error range curve of devices A-D.

SI-3. *J-V-L* and *CE-L-EQE* characteristics of devices.

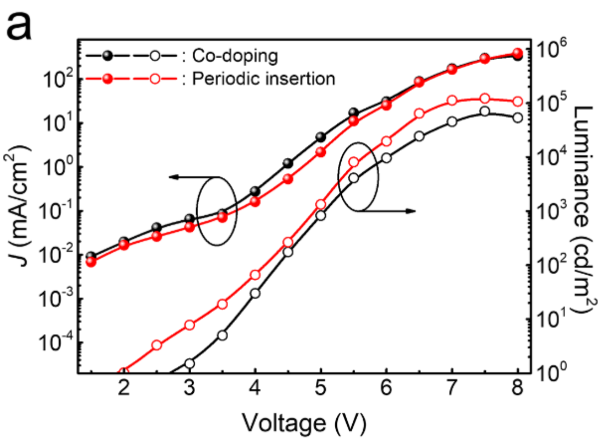

Figure S3. a) *J-V-L*, b) *CE-L-EQE* curves of the devices with HTLs structure of CBP:3.8 vol.% MoO_3 and CBP (16.7 nm)/MoO_3 (1 nm)/CBP (16.7 nm)/MoO_3 (1 nm)/CBP (16.7 nm).

SI-4. Energy band diagrams for devices at the equilibrium state.

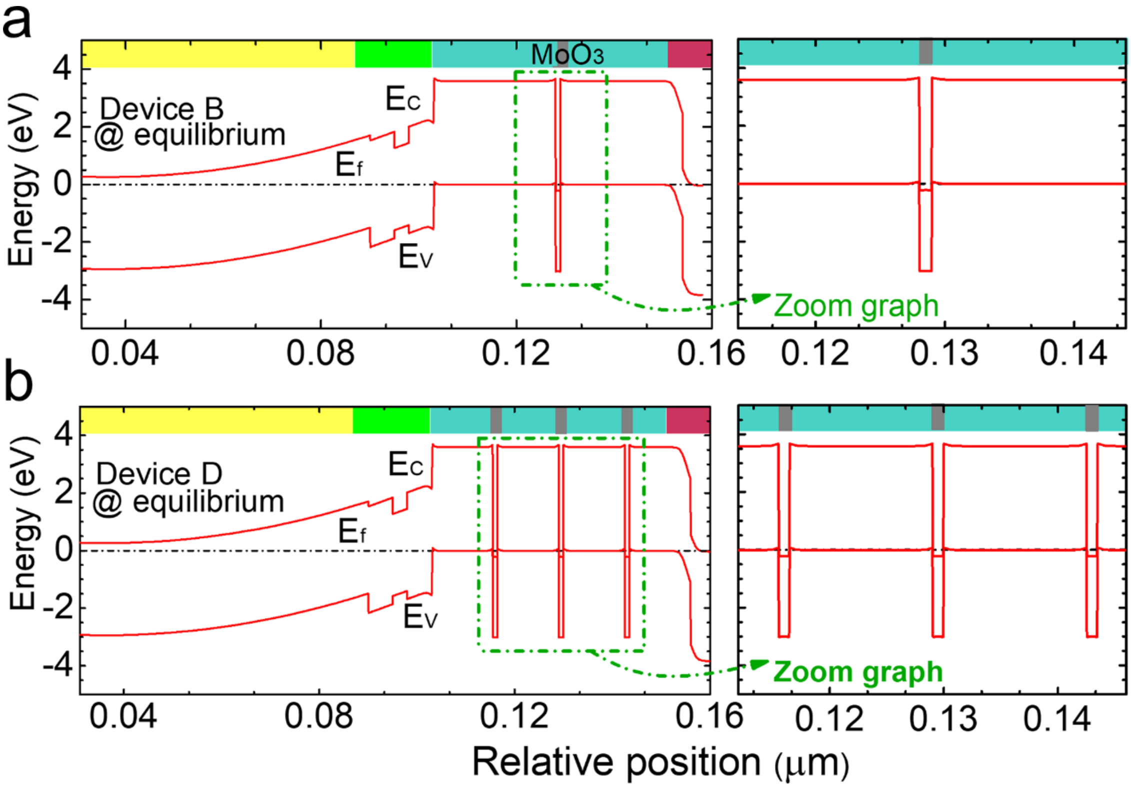

Figure S4. Energy band diagrams for a) Device B and b) Device D at the equilibrium state, respectively. E_c, E_v and E_f denote the conduction band, the valance band and quasi-Fermi level, respectively.

SI-5. AFM images of CBP films with/without periodic MoO_3 thin layer.

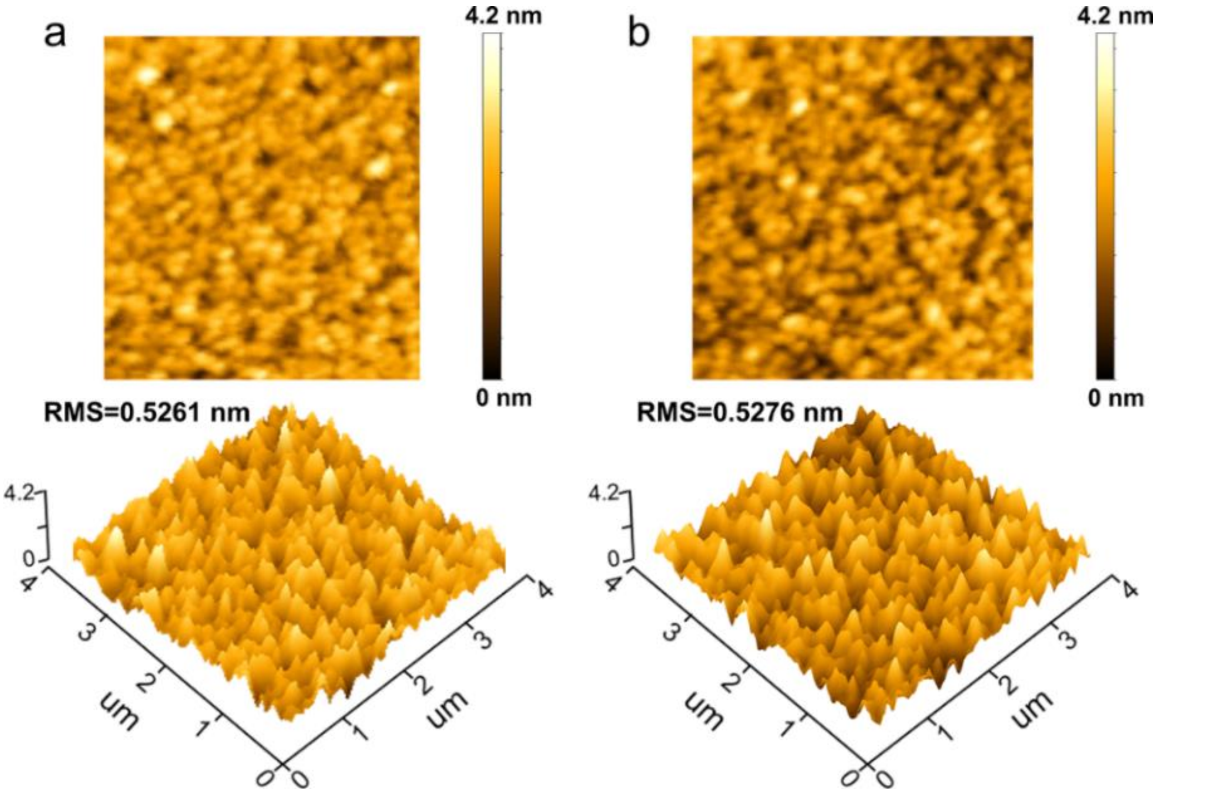

Figure S5. Surface morphology of a) the CBP film and b) CBP film with two periodic MoO_3 thin layer.

Table S1. Carrier mobility and carrier concentration parameters used in the simulations.

Electrical parameter	ITO (Ref)	ZnO (Ref)	CBP (Ref)	MoO_3 (Ref)	HTL (Ref)
Hole mobility [$cm^2V^{-1}s^{-1}$]	-	-	3.8×10^{-4} [4]	1.6×10^{2} [5]	-
Electron mobility [$cm^2V^{-1}s^{-1}$]	10^{2} [1]	1.8×10^{-3} [2]	-	-	3.3×10^{-2} [6]
Carrier concentration [cm^{-3}]	10^{20} [1]	10^{18} [3]	10^{1} [4]	10^{19} [5]	10^{19} [7]

References

[1] Z. Chen, Y. Zhuo, W. Tu, Z. Li, X. Ma, Y. Pei, G. Wang, *Opt. Express* **2018**, *26*, 22123.

[2] X. Dai, Z. Zhang, Y. Jin, Y. Niu, H. Cao, X. Liang, L. Chen, J. Wang, X. Peng, *Nature* **2014**, *515*, 96.

[3] J. H. Kim, E. M. Kim, D. Andeen, D. Thomson, S. P. DenBaars, F. F. Lange, *Adv. Funct. Mater.* **2007**, *17*, 463.

[4] J. C. Bolinger, L. Fradkin, K.-J. Lee, R. E. Palacios, P. F. Barbara, *P. Natl. Acad. Sci. USA* **2009**, *106*, 1342.

[5] M. Kovendhan, D. P. Joseph, P. Manimuthu, S. Sambasivam, S. N. Karthick, K. Vijayarangamuthu, A. Sendilkumar, K. Asokan, H. J. Kim, B. C. Choi, C. Venkateswaran, R. Mohan, *Appl. Surf. Sci.* 2013, *284*, 624.

[6] W. S. Jeon, J. S. Park, L. Li, D. C. Lim, Y. H. Son, M. C. Suh, J. H. Kwon, *Org. Electron.* **2012**, *13*, 939.

[7] S. Lee, J.-H. Lee, K. H. Kim, S.-J. Yoo, T. G. Kim, J. W. Kim, J.-J. Kim, *Org. Electron.* **2012**, *13*, 2346.
